# Diphtheria

**Published:** 1860-09

**Authors:** J. R. Jay

**Affiliations:** Chariton, Iowa


### DIPHTHERIA.

CHARITON, Iowa, Aug. 13, 1860.

*Editors Chicago Medical Journal:*

We have, for a time, been in a most destructive epidemic of diphtheria—destructive, because treated improperly, or not treated at all. It is confined to one township, on the western line of the county, ordinarily one of our most healthy neighborhoods. In a sparsely settled country, it leaped across rolling, unimproved prairies, attacking, with great virulence, whole families who had hitherto been strangers to disease.

If called when the yellowish white spots first make their appearance on the tonsils, I have always succeeded in checking it; but have found great difficulty in successfully treating cases of some days' standing.

I have used, with good effect a sat'd solution of nitrate of silver to the tonsils, &c. I have used quinine and chlorate of potash plentifully. I prefer the mur. tinc. of iron to the potash. I have used mercury sparingly but have seen no bad effect from it.

When the hurry is over I may give you some particulars.

Yours, &c., J. R. Jay.

